# Impact of combined vector control interventions on *Anopheles gambiae *sensu lato resistance dynamics in a high pyrethroid resistance settings in Southwestern Burkina Faso

**DOI:** 10.1186/s12936-025-05702-1

**Published:** 2025-12-05

**Authors:** Kelly L. Ngaffo, Dieudonné D. Soma, Aristide S. Hien, Karama O. Delphine, Samina Maiga, Didier P. Alexandre Kaboré, Bamogo Rabila, Moussa Namountougou, Abdoulaye Diabaté, Etang D. Josiane, Roch K. Dabiré

**Affiliations:** 1 Institut de Recherche en Sciences de La Santé, Direction Régionale de l’ouest, BP 545, Bobo-Dioulasso, Burkina Faso; 2 Université Nazi Boni, Bobo-Dioulasso, Burkina Faso; 3 Laboratoire de Recherche sur le Paludisme, Institut de Recherche de Yaoundé, Organisation de Coordination pour la lutte Contre les Endémies en Afrique Centrale, Yaoundé, Cameroun

**Keywords:** Insecticide resistance, Resistance monitoring, Integrated vector management, PBO-treated nets, Burkina Faso

## Abstract

**Background:**

The widespread emergence of insecticide resistance in malaria vectors, particularly *Anopheles gambiae*, poses a significant challenge to malaria control in sub-Saharan Africa. In Burkina Faso, where pyrethroid resistance is driven by both target-site mutations and enhanced metabolic detoxification, the efficacy of key vector control tools such as insecticide-treated nets (ITNs) and indoor residual spraying (IRS) has declined. In response, alternative non-pyrethroid interventions are being implemented. This study investigated the impact of non-pyrethroid IRS and piperonyl butoxide (PBO)-synergist pyrethroid ITNs on insecticide resistance dynamics in *An. gambiae* populations in Kampti and Gaoua, southwestern Burkina Faso.

**Methods:**

*Anopheles gambiae* larvae were collected annually from 2021 to 2023, between the months of August and September. Adult susceptibility to pyrethroids insecticides was assessed using WHO tube assays. Molecular assays were used for species identification within the *An. gambiae* complex and detection of the *kdr* mutations. Expression of metabolic resistance genes was quantified using qPCR. KD60 and mortality rates were compared across intervention: during IRS use (2021), after its replacement with PBO-ITNs (2022), and after 2 years of PBO-ITN implementation (2023).

**Results:**

Three *Anopheles* species were identified, 397 *An. gambiae *sensu stricto (66.17%; 95%CI 62.30–69.80) 149 *Anopheles arabiensis* (; 24.83%; 95%CI 21.50–28.40) and 54; *Anopheles coluzzii* (9%; 95CI 6.96–11.60). with *An. gambiae* being predominant. The L1014F mutation was highly prevalent, while L1014S was undetected. Following the withdrawal of IRS and the introduction of PBO-ITNs in 2022, pyrethroid resistance intensified in Kampti, with reduced mortality to permethrin and alphacypermethrin. PBO pre-exposure partially restored deltamethrin susceptibility in Kampti and fully restored it in Gaoua. By 2023, resistance remained high, particularly to alphacypermethrin, and PBO’s synergistic effect diminished. Overexpression of CYP6M2 and CYP6Z1 was consistently observed, suggesting enhanced metabolic resistance.

**Conclusion:**

The findings indicated that while PBO-ITNs initially improved vector susceptibility to pyrethroids, their efficacy declined over time. This study underscores the dynamic nature of insecticide resistance and highlights the need for sustained resistance monitoring and diversified vector control strategies in high-resistance settings like southwestern Burkina Faso.

**Supplementary Information:**

The online version contains supplementary material available at 10.1186/s12936-025-05702-1.

## Background

Vector control remains the cornerstone of malaria prevention strategies in sub-Saharan Africa, where it has significantly contributed to reducing transmission over the past two decades [1, 2]. However, the increasing prevalence of insecticide resistance among malaria vector populations particularly *Anopheles gambiae *sensu lato (*s.l.*) now threatens the long-term effectiveness of these interventions [3, 4]. Pyrethroids, the only insecticide class currently approved for use in insecticide-treated nets (ITNs) and a common choice for indoor residual spraying (IRS), are of particular concern due to widespread resistance [5–7]. First reported in *An. gambiae s.l.* from Côte d’Ivoire in 1993 [8], pyrethroid resistance has since expanded across much of sub-Saharan Africa, with West Africa and Burkina Faso among the most affected regions [1, 9, 10].

This resistance has been largely attributed to sustained selection pressure from intensive and prolonged use of insecticides in both public health and agriculture [11–14]. Two primary mechanisms underlie resistance in malaria vectors: target-site mutations in the voltage-gated sodium channel gene and metabolic resistance via upregulation of detoxification enzymes [15, 16]. In target site resistance, the mosquito becomes resistant by modifying the target site of the insecticide, preventing it from binding effectively, so that the insecticide has little or no effect on the insect. Metabolic resistance, characterized by the degradation of the insecticide into less toxic or non-toxic products, thus reducing the quantity of toxic molecules reaching the target. In Burkina Faso, resistance to pyrethroids is now established nationwide [17]. Molecular studies have revealed the widespread presence of knockdown resistance (*kdr*) mutations, particularly Vgsc-1014F and, to a lesser extent, 1014S, as well as the N1575Y mutation [9, 17–19]. In addition, elevated expression of detoxifying enzymes, such as cytochrome P450 monooxygenases (e.g., CYP6M2, CYP6Z1) and glutathione S-transferases, has been implicated in conferring metabolic resistance [18–20]. Despite growing evidence, the contribution of many resistance-associated genes remains underexplored in several regions, including southwestern Burkina Faso.

In response to the rising threat of resistance, the World Health Organization (WHO) has recommended diversifying vector control strategies through the introduction of novel insecticide classes and formulations, including those combining pyrethroids with synergists such as piperonyl butoxide (PBO) or alternative compounds for IRS [21]. Between 2018 and 2021, the President’s Malaria Initiative (PMI) supported IRS campaigns in three regions of Burkina Faso Centre-Nord, Boucle du Mouhoun, and Sud-Ouest alongside repeated mass ITN distribution campaigns. The insecticides used for the IRS were pirimiphos-methyl, clothianidin, and a combination of clothianidin and deltamethrin, which belong respectively to the organophosphate, neonicotinoid, and neonicotinoid + pyrethroid classes. The ITNs consisted of conventional LLINs, PBO and IG2 nets. [17, 22, 23]. Although these efforts aimed to mitigate the impact of pyrethroid resistance, their influence on vector susceptibility remains uncertain. Studies across various endemic countries have suggested that such interventions may inadvertently intensify resistance and contribute to malaria resurgence.

The Southwest region of Burkina Faso is a high-transmission zone with nearly perennial malaria transmission. It is characterized by considerable vector species diversity and intense levels of insecticide resistance in *An. gambiae* s*.l.*, particularly linked to the high prevalence of *kdr* mutations [17, 19, 20, 22]. As a result, this region has emerged as a resistance hotspot, where conventional tools such as ITNs and IRS are increasingly compromised. The persistence of high-intensity resistance in this area threatens to undermine existing vector control strategies and jeopardize malaria control progress.

To ensure the continued effectiveness of interventions, WHO advocates for data-driven decision-making adapted to local epidemiological contexts [24], while integrated vector management (IVM) promotes coordinated, evidence-based use of resources for sustainable control [25]. However, such approaches require robust, context-specific entomological data to guide intervention strategies.

In this context, the present study was undertaken in the districts of Gaoua and Kampti in southwestern Burkina Faso. It aimed to assess the impact of combined vector control interventions including IRS and PBO-pyrethroid ITNs on the insecticide susceptibility of local *An. gambiae* populations. The findings are intended to inform the National Malaria Control Programme (NMCP) in selecting and deploying effective, locally adapted interventions to address rising resistance and sustain vector control outcomes in high-risk areas.

## Methods

### Study area

**Fig. 1:**
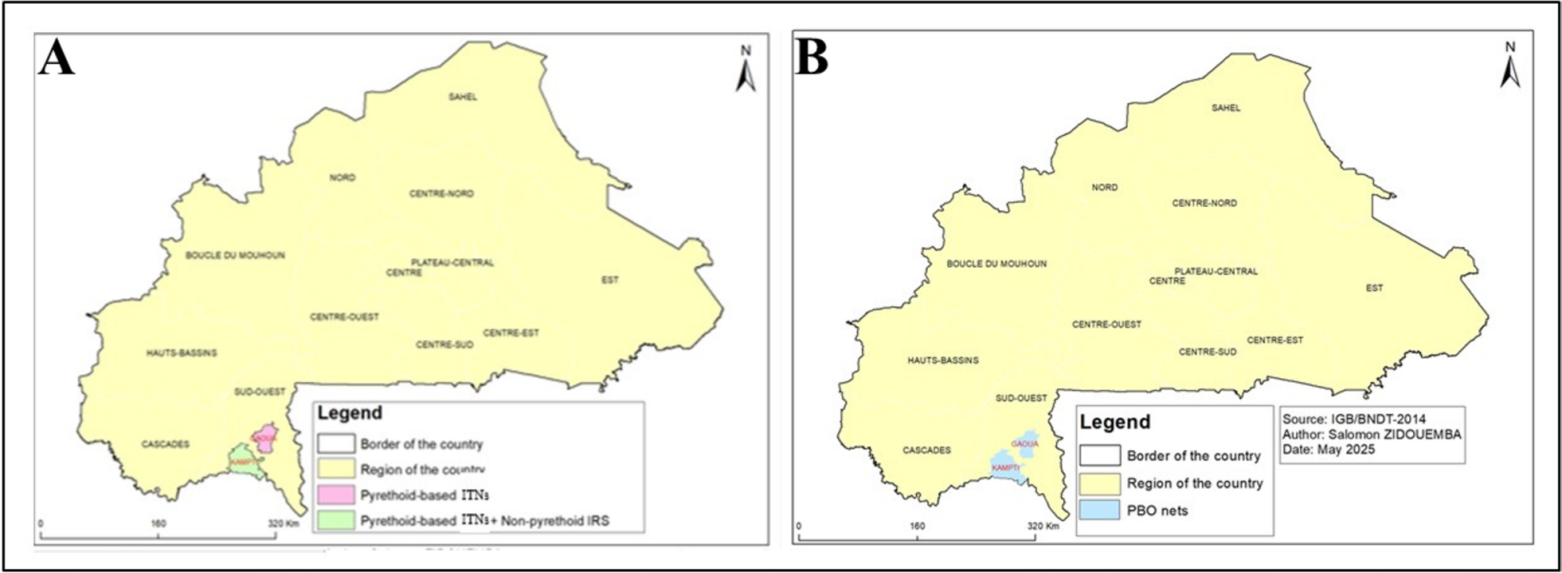
Study area and intervention timeline

This study was conducted in two districts of the Sud-Ouest administrative region of Burkina Faso: Gaoua (10°19′46″ N, 3°10′41″ W) and Kampti (10°07′60″ N, 3°27′00″ W) (Fig. 1). The region is situated within the Sudanian ecological zone and is characterized by a tropical climate with two well-defined seasons: a dry season extending from November to May, and a rainy season from May to October. The mean annual rainfall is approximately 1200 mm, and the average temperature hovers around 27 °C. The landscape comprises a mosaic of savanna vegetation, agricultural land, and a network of rivers, dams, and wetlands, with an average altitude of 450 m above sea level.

The local hydrographic system includes tributaries of major river basins and supports a variety of larval habitats conducive to *Anopheles* breeding. Agriculture is the primary economic activity, with crops such as cereals, cotton, peanuts, and tubers commonly cultivated. Residential structures are predominantly made of mud, typical of rural settings in the region.

Malaria transmission in the Sud-Ouest region is perennial, typically intensifying at the onset of the rainy season in May [22]. The region bears a high malaria burden, ranking second in incidence after the Sahel region, and exhibiting a malaria-attributable mortality rate of 33.8%. In recent years, several vector control interventions have been deployed in the study area. Between 2018 and 2021, the Kampti district received indoor residual spraying (IRS) based clothianidin (2018), deltamethin + clothianidin (2019), deltamethin + clothianidin (2020), pirimiphos-methyl (2021), in addition to standard insecticide-treated nets (ITNs) (PermaNet^®^ 2.0). Over the same period, Gaoua was covered exclusively by PermaNet^®^ 2.0 ITNs. In 2022, both districts transitioned to next-generation ITNs incorporating the synergist piperonyl butoxide such as DurNe^®^t Plus in Gaoua and Olyset^®^ Plus in Kampti), aimed at mitigating the impact of pyrethroid resistance.

### Study design

This study was designed to evaluate the impact of two malaria vector control strategies, insecticide-treated nets (ITNs: PermaNet^®^ 2.0 and PBO-nets) and indoor residual spraying (IRS) with non-pyrethroid insecticides (Actellic^®^ 300CS based pirimiphos-methyle), on the resistance profiles of *An. gambiae* populations in southwestern Burkina Faso. The investigation was conducted across a 3-year period (2021–2023) and focused on two districts: Kampti and Gaoua, which had distinct intervention histories.

The study had three primary objectives: (i) to compare vector resistance parameters namely, knockdown at 60 min (KD₆₀) and 24-h mortality between Kampti, where IRS with the organophosphate insecticide pirimiphos-methyl was implemented, and Gaoua, which was covered by pyrethroid-only ITNs (PermaNet^®^ 2.0) in 2021; (ii) to assess changes in resistance parameters in 2022 following the introduction of next-generation ITNs containing a combination of pyrethroids and the synergist piperonyl butoxide (PBO) in both sites, replacing IRS in Kampti and pyrethroid-only ITNs in Gaoua; (iii) to evaluate the impact of two consecutive years of PBO-pyrethroid ITN deployment (2022–2023) on both phenotypic resistance (KD₆₀ and mortality) and metabolic resistance, through the quantification of gene expression associated with detoxification pathways.

These sequential comparisons allowed for the assessment of how different control tools and their combinations influence resistance dynamics over time. The timing and distribution of the interventions across the study period are illustrated in Fig. 1. By combining bioassay data with molecular analyses, this study aimed to provide actionable insights into the efficacy and long-term sustainability of non-pyrethroid-based strategies in areas burdened by high levels of pyrethroid resistance.

### Larval collections

Larvae of *An. gambiae s.l.* were collected during the peak malaria transmission season, between August and September, in 2021, 2022, and 2023. Sampling was conducted at *An. gambiae s.l.* larval habitats, including drainage ditches, puddles, stream-bed pools, and swamps, located within the study sites of Kampti and Gaoua. These habitats were systematically surveyed, and larvae were collected using standard dipping techniques.

Following collection, larvae were transported to the insectary at the Institut de Recherche en Sciences de la Santé (IRSS) in Bobo-Dioulasso. In the insectary, specimens were reared to adulthood under controlled environmental conditions: temperature maintained at 27 ± 3 °C, relative humidity at 60–80%, and a 12:12 h light:dark photoperiod. Adult female mosquitoes aged 2–5 days and not previously blood-fed were subsequently selected for use in insecticide susceptibility bioassays and molecular analyses.

### Susceptibility and resistance intensity assays of pyrethroids insecticides

To assess the phenotypic resistance profile of *An. gambiae* populations in Gaoua and Kampti in relation to vector control interventions, insecticide susceptibility tests were conducted over three consecutive years (2021, 2022, and 2023). These assays were carried out following World Health Organization (WHO) guidelines using standard diagnostic concentrations of three pyrethroid insecticides: 0.05% alpha-cypermethrin, 0.05% deltamethrin, and 0.75% permethrin [26].

In addition to susceptibility testing, resistance intensity assays were performed in 2022 and 2023 to determine the strength of resistance in mosquito populations. These tests employed increasing concentrations of each insecticide at 1 × (diagnostic dose), 5 ×, and 10 ×, as recommended by WHO protocols. For each concentration and insecticide, adult female mosquitoes were exposed for 60 min in WHO test tubes, with four replicates per treatment group (20–25 mosquitoes per replicate). Mortality was recorded 24 h post-exposure. Negative controls (wild *An. gambiae s.l.* from the same populations) were included in each assay using two replicates of test tubes lined with filter paper treated with silicone oil only, to account for handling mortality. The insecticide-susceptible *An. gambiae *sensu stricto (*s.s*.) Kisumu laboratory strain was used as a positive control to validate the efficacy of the test papers.

### PBO synergist bioassay

To evaluate the ability of piperonyl butoxide (PBO) to restore susceptibility to pyrethroids in *An. gambiae* populations with high levels of resistance, synergist bioassays were performed on mosquitoes collected from Gaoua and Kampti. Female mosquitoes aged 2–5 days, unfed and reared under controlled insectary conditions, were pre-exposed for 1 h to 4% PBO-impregnated papers, following WHO standard procedures [26].

Immediately following pre-exposure, mosquitoes were exposed for 60 min to one of three pyrethroid insecticides at diagnostic concentrations: 0.75% permethrin, 0.05% deltamethrin, or 0.05% alpha-cypermethrin. Each treatment was conducted in four replicates, and mortality was assessed 24 h post-exposure. These assays were conducted annually over a 3-year period (2021, 2022, and 2023) to monitor temporal changes in PBO efficacy and to assess the potential role of metabolic resistance mechanisms in mediating pyrethroid resistance.

### Molecular analysis

#### Molecular species identification and genotyping of Vgsc-1014F and Vgsc-1014S

Genomic DNA was extracted from the head-thorax of both dead and surviving *An. gambiae s.l.* specimens collected during insecticide bioassays between 2021 and 2023, using a 2% CetylTrimethyl Ammonium Bromide (CTAB) extraction protocol [27]. Species within the *An. gambiae* complex were identified by polymerase chain reaction (PCR) employing the SINE200 assay as described by Santolamazza et al*.* [28].

Genotyping of the knockdown resistance mutations Vgsc-1014F and Vgsc-1014S in the voltage-gated sodium channel gene was performed using allele-specific PCR according to the protocols established by Martinez-Torres et al*.* [29]. These assays allowed for the detection and frequency estimation of the target-site mutations associated with pyrethroid resistance in the vector populations.

#### Metabolic resistance assays

To further elucidate the mechanisms underlying insecticide resistance observed in 2023, we quantified the expression levels of key detoxification enzymes implicated in pyrethroid resistance. After the susceptibility tests, the negative control mosquitoes were chilled for approximately five minutes to immobilize them, then preserved in 2 mL tubes containing 1.5 mL of RNAlater, with ten mosquitoes per tube. Mosquitoes were placed in the tubes so that they were completely immersed in RNAlater to ensure proper RNA preservation and stored at − 20 °C until RNA extraction. Prior to RNA extraction, the excess RNAlater was removed by gently blotting the mosquitoes on absorbent paper. For each study site, 50 *An. gambiae s.l.* specimens, originating from the negative controls of the bioassays, were individually used for RNA extraction together with samples from the insecticide-susceptible *An. gambiae s.s.* Kisumu laboratory colony.

Total RNA was isolated using the RNeasy^®^ Mini Kit (QIAGEN) following the manufacturer’s protocol and subsequently reverse transcribed into complementary DNA (cDNA) using the FIREScript^®^ RT cDNA Synthesis Kit (SOLIS BIODYNE). RNA and cDNA concentrations and purity were assessed spectrophotometrically prior to downstream analysis.

Quantitative real-time PCR (qRT-PCR) was performed to measure the relative expression of six genes associated with metabolic resistance: cytochrome P450 monooxygenases *CYP6AG2*, *CYP6M2*, *CYP6P1*, *CYP6L1*, *CYP6Z1*, and the glutathione S-transferase *GSTe2*. Gene expression was quantified using primers and protocols described by Nardini et al*.* [30] and *β-actin* used as the reference gene.

Each reaction was run in triplicate with a total volume of 10 µl, comprising 3 µl of 5 × HOT FIREPol^®^ EvaGreen^®^ qPCR Mix Plus (ROX), 0.4 µl of primer (10 µM), 1 µl of cDNA, and nuclease-free water. Amplification was conducted on a QuantStudio^™^ 1 Real-Time PCR System (Applied Biosystems^™^, Waltham, MA, USA) using the following cycling conditions: initial denaturation at 95 °C for 15 min, followed by 40 cycles of 95 °C for 15 s, annealing at 60 °C (or 58 °C) for 30 s, and extension at 72 °C for 30 s.

### Data analysis

Data were processed and analyzed using Microsoft^®^ Excel^®^ 2016 (MSO Version 2206 Build 16.0.15330.20216) and R software version 4.1.3. Bioassay results were interpreted based on percentage mortality, with standard error of the mean (SEM) calculated for each estimate. Where necessary, mortality rates were corrected according to World Health Organization (WHO) guidelines [26]. For the insecticide susceptibility assay, a mortality in the range of 98–100% indicates susceptibility of the mosquitoes; a mortality rate between 90 and 97% is suggestive of possible resistance that needs to be confirmed and a mortality less than 90% confirmed the existence of resistance. For the effect of pre-exposure to the synergist PBO on mosquito susceptibility, WHO recommendations were also use. If mean mortality in the “insecticide only” samples is ≥ 90%, the effect of PBO cannot be reliably assessed. If the mean mortality in the “insecticide only” samples is < 90%, the effect of PBO can be interpreted according to the following criteria:

- Complete restoration of susceptibility (mitigation of resistance) by pre-exposure to PBO (i.e. ≥ 98% mean mortality in the “PBO followed by insecticide” samples) implies that a monooxygenase-based resistance mechanism fully accounts for expression of the resistant phenotype in the test population
- Partial restoration of susceptibility by pre-exposure to PBO (i.e. mean mortality in the “PBO followed by insecticide” samples is greater than mean mortality in the “insecticide only” samples but < 98%) implies that a monooxygenase-based resistance mechanism only partially accounts for expression of the resistant phenotype and that other resistance mechanisms are likely to be present in the test population.
- No restoration of susceptibility by pre-exposure to PBO (mean mortality in the “PBO followed by insecticide” samples is equal to or lower than mean mortality in the “insecticide only” samples) implies that the resistance phenotype detected is not based on monooxgenase-mediated detoxification.

Gene expression levels were quantified using the comparative threshold cycle (Ct) method (2-ΔCT), normalizing target gene expression to a housekeeping reference gene (*β-actin*) to enable relative comparisons across samples. Statistical differences in gene expression between study sites were evaluated using the Wilcoxon rank-sum test. The Wilcoxon rank-sum test was chosen instead of the t-test because the sample sizes were small and the data did not meet the normality assumptions required for the t-test. Differences in categorical variables, such as mortality rates and genotype frequencies, were assessed with Chi-square or Fisher’s exact tests, as appropriate. Chi-square tests were used to compare the mean mortality rates because the sample sizes were sufficiently large and the expected frequencies met the assumptions of the test. Fisher’s exact tests were applied to compare proportions when some groups had small sample sizes with expected counts below five, making the Chi-square test inappropriate. A significance threshold of p ≤ 0. 05 was applied throughout all analyses to determine statistical significance.

## Results

### Species composition of the *Anopheles gambiae* complex and distribution of Vgsc-L1014F and L1014S alleles over the study period

#### *Anopheles gambiae* species composition

**Fig. 2:**
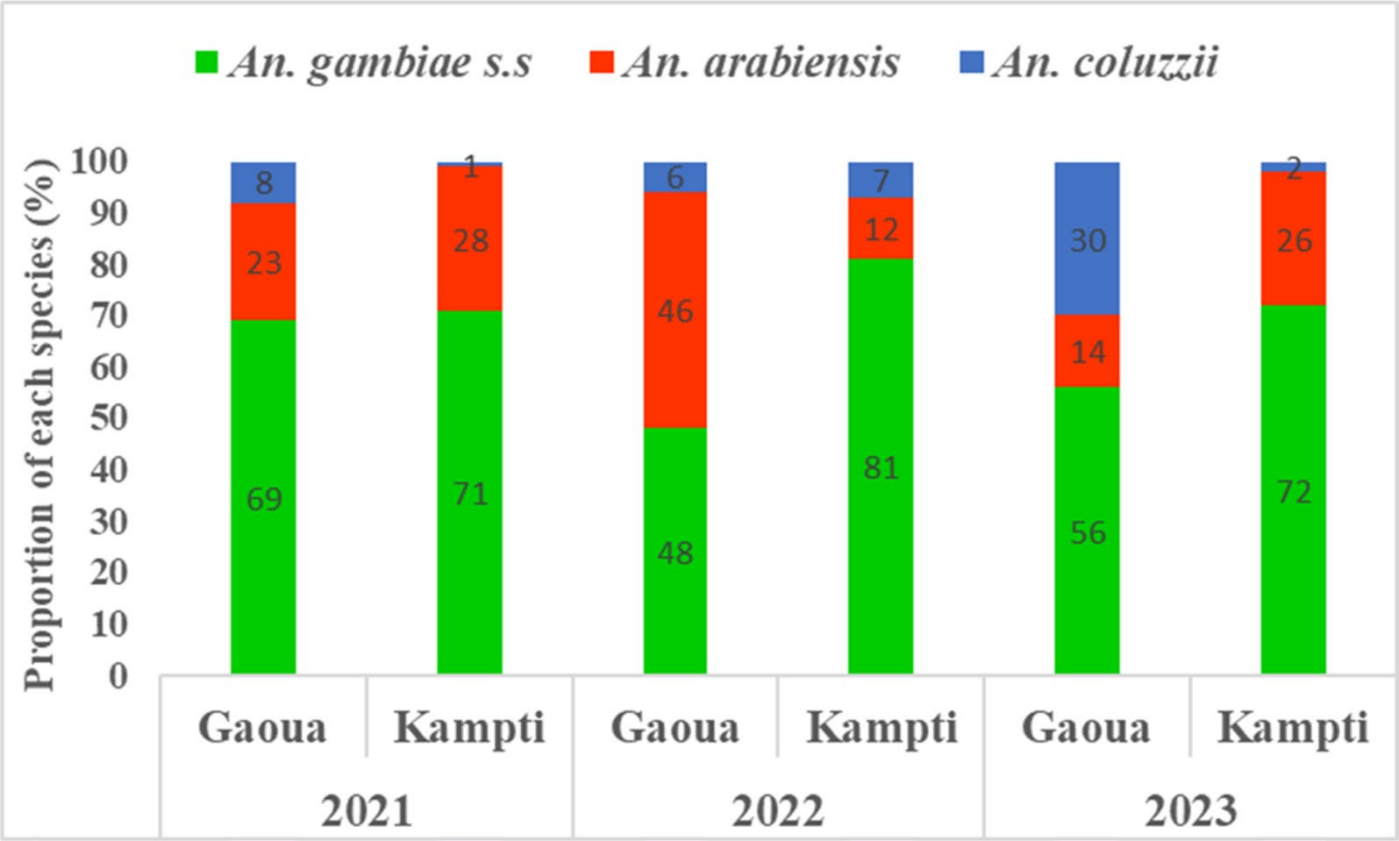
Species composition of the *Anopheles gambiae* complex across study sites over 3 years, determined by PCR analysis

A total of 300 mosquitoes (100 per site per year from deltamethrin susceptibility test) were analyzed by PCR to determine species composition within the *An. gambiae* complex (Fig. 2). Three species were identified: *An. gambiae s.s*., *Anopheles arabiensis*, and *Anopheles coluzzii*. Across all study sites and years, *An. gambiae s.s.* was the predominant species, comprising 48–81% of the populations sampled. *Anopheles arabiensis* was the second most abundant species, representing 12–46%, while *An. coluzzii* was generally the least frequent, ranging from 1 to 30%. An exception was observed in Gaoua in 2023, where *An. coluzzii* (30%) surpassed *An. arabiensis* in relative abundance (Supplementary File 1).

#### Genotyping of Vgsc-L1014F and L1014S target site mutations

The frequencies and distribution of the *Vgsc* L1014F and L1014S mutations within *An. gambiae* populations are summarized in Table 1. The L1014F mutation was detected across all species of the *An. gambiae* complex, with variable frequencies depending on species, site, and year. In 2021, the L1014F allele frequency was 0.58 in *An. gambiae s.s.* from Kampti and 0.52 in Gaoua; for *An. arabiensis*, frequencies were 0.44 in Kampti and 0.80 in Gaoua; while *An. coluzzii* exhibited frequencies of 0.50 in Kampti and 0.41 in Gaoua. In 2022, L1014F frequencies remained similar, with *An. gambiae s.s.* showing 0.53 in Kampti and 0.54 in Gaoua, and *An. arabiensis* presenting frequencies of 0.50 and 0.46 in Kampti and Gaoua, respectively. By 2023, *An. gambiae s.s.* populations exhibited increased L1014F frequencies ranging from 0.77 in Kampti to 0.83 in Gaoua. *An. arabiensis* showed frequencies between 0.50 (Gaoua) and 0.77 (Kampti), whereas *An. coluzzii* maintained a consistent frequency of approximately 0.50 across both sites (Supplementary File 2).

**Table 1:** Allelic frequencies of Vgsc-L1014F and Vgsc-L1014S mutations in *Anopheles gambiae* populations from Gaoua and Kampti (2021–2023)

Year	Site	Species	N	L1014L	L1014F	L1014S	L1014F	L1014L	L1014L	Frequencies	
				L1014L	L1014F	L1014S	L1014S	L1014F	L1014S	L1014F	L1014S
2021	Gaoua	*An. arabiensis*	15	3	0	0	0	12	0	0.80	0
		*An. coluzzii*	6	1	0	0	0	5	0	0.41	0
		*An. gambiae s.s*	29	1	2	0	2	24	0	0.52	0.03
	Kampti	*An. arabiensis*	9	1	0	0	0	8	0	0.44	0
		*An. coluzzii*	1	0	0	0	0	1	0	0.50	0
		*An. gambiae* *s.s.*	40	0	6	0	1	33	0	0.58	0.01
2022	Gaoua	*An. arabiensis*	38	7	4	0	1	26	0	0.46	0.01
		*An. gambiae*	12	1	2	0	1	8	0	0.54	0.04
	Kampti	*An. arabiensis*	1	0	0	0	0	1	0	0.5	0
		*An. gambiae* *s.s.*	47	3	6	0	2	36	0	0.53	0.02
2023	Gaoua	*An. arabiensis*	7	2	2	0	0	3	0	0.50	0
		*An. coluzzii*	13	2	2	0	1	8	0	0.50	0.04
		*An. gambiae* *s.s.*	30	0	20	0	0	10	0	0.83	0
	Kampti	*An. arabiensis*	13	0	7	0	0	6	0	0.77	0
		*An. coluzzii*	1	0	0	0	0	1	0	0.50	0
		*An. gambiae* *s.s*.	36	0	20	0	4	12	0	0.77	0.06

Statistical analyses revealed no significant temporal changes in L1014F allele frequencies between 2021 and 2022 or between 2022 and 2023 within each site (p > 0.5), indicating stable prevalence over the study period. In contrast, the L1014S mutation was detected at markedly lower frequencies across all species, with a maximum frequency of 0.06 observed, suggesting that this mutation remains relatively rare in the populations studied.

#### Shifts in *Anopheles gambiae* resistance in Kampti following the replacement of IRS and standard ITNs with PBO-pyrethroid nets in southwestern Burkina Faso between 2021 and 2022

Between 2021 and 2022, mean mortality rates of *An. gambiae s.l.* from Kampti following 60-min exposure to permethrin and alpha-cypermethrin significantly declined, including after pre-exposure to PBO followed by these insecticides (Table 2). The highest mean KD₆₀ with a single insecticide was observed in 2021 for alpha-cypermethrin (13.45%), while the lowest KD₆₀ rates were recorded in 2022 for both permethrin and alpha-cypermethrin (0%). No significant difference in KD₆₀ rates was detected for deltamethrin between the 2 years (χ^2^ = 1.1063, df = 1, p = 0.293).

**Table 2:** Mortality rate of *Anopheles gambiae* s.l. in Kampti after 60 min of exposure to pyrethroid only and pre-exposure to PBO in 2021 and 2022

Insecticide	Year	Mean KD60 (SEM)	Chi square (χ2)
Permethrin 0.75%	2021	15.78 (± 1.90)	0.0001
	2022	0.00 (± 0.00)	
Permethrin 0.75% + PBO	2021	31.10 (± 4.03)	3.272e-08
	2022	0.00 (± 0.00)	
Deltamethrin 0.05%	2021	12.17 (± 2.40)	0.2929
	2022	6.35 (± 1.73)	
Deltamethrin 0.05% + PBO	2021	94.11 (± 2.84)	2.2e-16
	2022	6.65 (± 2.33)	
Alphacypermethrin 0.05%	2021	13.45 (± 3.66)	0.00074
	2022	0.00 (± 0.00)	
Alphacypermethrin 0.05% + PBO	2021	91.75 (± 1.31)	2.2e-16
	2022	0.00 (± 0.00)	

**Fig. 3:**
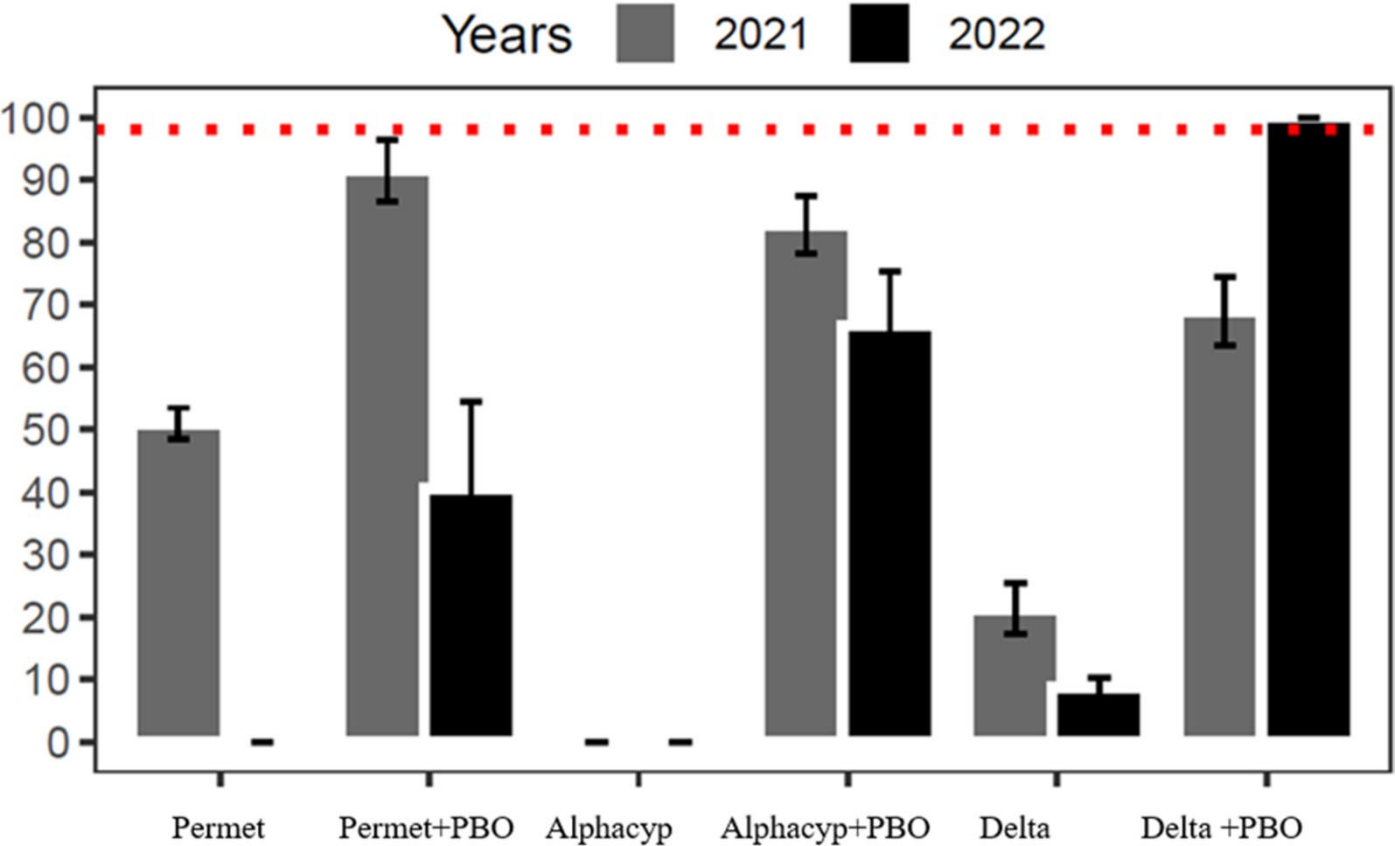
Twenty-four-hour mortality rates of *Anopheles gambiae s.l.* from Kampti in WHO tube tests with permethrin (0.75%), alpha-cypermethrin (0.05%), and deltamethrin (0.05%) with and without PBO pre-exposure in 2021 and 2022

WHO tube bioassays conducted with pyrethroids and pyrethroids combined with 4% PBO on *An. gambiae* populations from Kampti in 2021 and 2022 confirmed resistance (mean mortality < 60%) to all three tested pyrethroids (Fig. 3). Mortality rates declined for all insecticides between 2021 and 2022 after 24 h of observation, with significant reductions observed for permethrin and deltamethrin. Specifically, mortality for permethrin dropped sharply from 50.96% in 2021 to 0% in 2022, while deltamethrin mortality decreased from 21.30% to 8.69% (P < 0.03). Pre-exposure to 4% PBO prior to pyrethroid exposure significantly increased mortality rates across all insecticides tested (P < 0.05) in Kampti. Notably, PBO pre-exposure fully restored susceptibility to deltamethrin in 2022. However, susceptibility was only partially restored for alpha-cypermethrin and permethrin following PBO pre-exposure. Furthermore, the mortality rate after PBO pre-exposure with permethrin was significantly lower in 2022 compared to 2021 (P < 0.02).

Knockdown rates for pyrethroids deltamethrin (0.05%), alpha-cypermethrin (0.05%), and permethrin (0.75%) and pyrethroids combined with PBO exhibited significant variation between 2021 and 2022 in Gaoua (p < 0.01) (Table 3). An exception was observed for deltamethrin, where no statistically significant difference in knockdown rates was found between the 2 years (χ^2^ = 3.6243, df = 1, p = 0.057), despite a decrease in mortality rates after 60 min of exposure from 37.07% in 2021 to 22.64% in 2022.

**Table 3:** Mortality of *Anopheles gambiae* *s.l.* following 60-min exposure to pyrethroids with and without PBO pre-exposure in Gaoua, 2021–2022

Insecticide	Year	Mean KD60 (SEM)	Chi square (χ2)
Permethrin 0.75%	2021	17.68 (± 2.34)	0.00309
	2022	2.38 (± 2.38)	
Permethrin 0.75% + PBO	2021	34.06 (± 4.67)	3.622e-08
	2022	2.17 (± 2.17)	
Deltamethrin 0.05%	2021	37.07 (± 2.74)	0.05694
	2022	22.64 (± 5.82)	
Deltamethrin 0.05% + PBO	2021	98.86 (± 1.13)	2.2e-16
	2022	22.13 (± 5.51)	
Alphacypermethrin 0.05%	2021	12.42 (± 4.43)	2.2e-16
	2022	1.00 (± 1.00)	
Alphacypermethrin 0.05% + PBO	2021	84.50 (± 3.01)	2.2e-16
	2022	1.25 (± 1.25)	

**Fig. 4:**
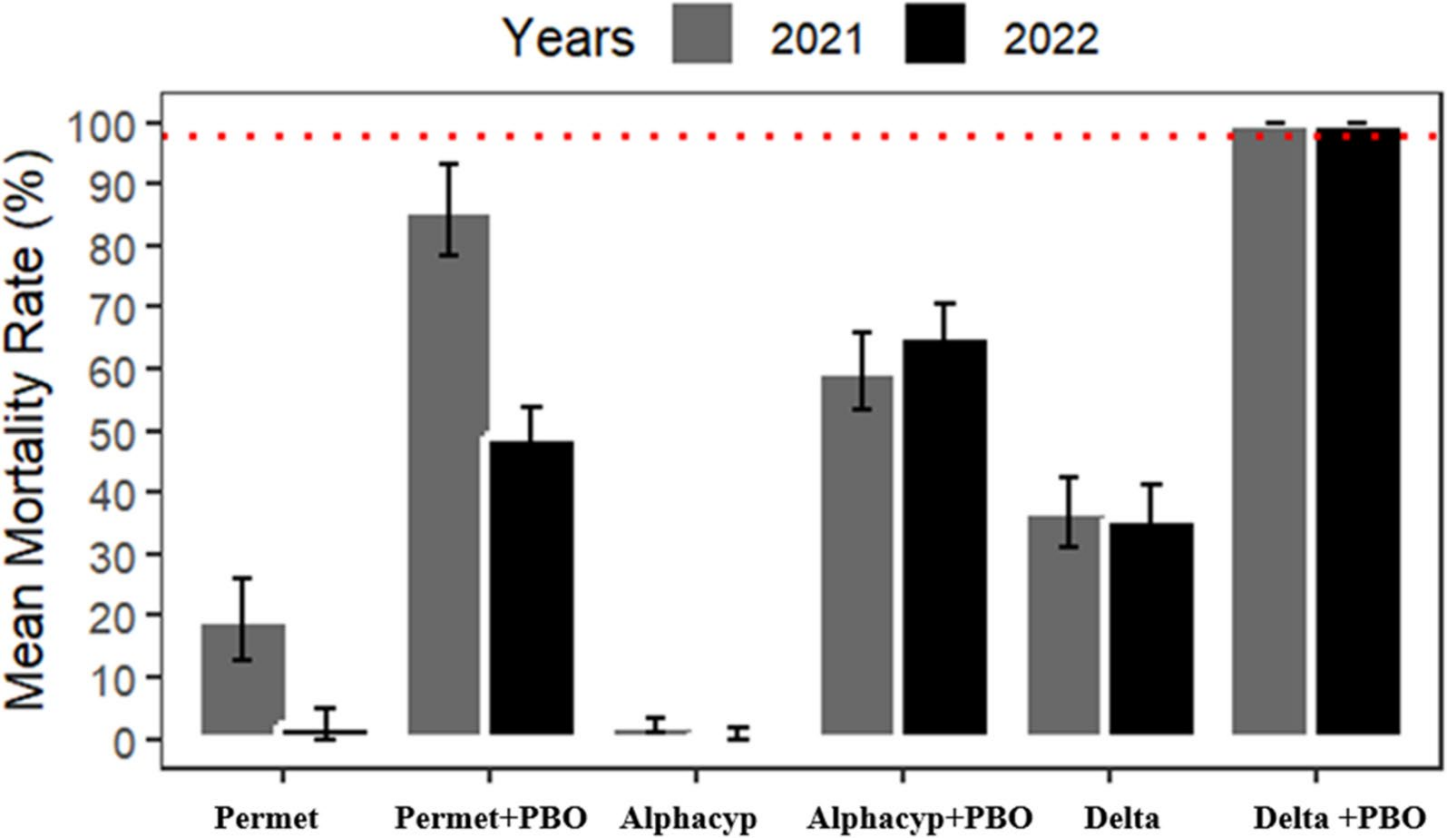
Twenty-four-hour mortality of *Anopheles gambiae s.l.* in Gaoua following WHO tube tests with permethrin (0.75%), alpha-cypermethrin (0.05%), and deltamethrin (0.05%), with and without PBO pre-exposure, in 2021 and 2022

Mean mortality rates of *An. gambiae s.l.* following exposure to pyrethroids in Gaoua remained below 40% in both 2021 and 2022 (Fig. 4). Overall, mortality rates declined across all tested insecticides between these years; however, a statistically significant reduction was detected only for permethrin (χ^2^ = 9.7141, df = 1, p = 0.0018). Pre-exposure to PBO prior to pyrethroid exposure restored full susceptibility exclusively for deltamethrin in both years. In contrast, permethrin and alpha-cypermethrin demonstrated only partial restoration of susceptibility following PBO pre-exposure, with mortality rates differing significantly between 2021 and 2022 (P < 0.0005).

### Resistance profile of *Anopheles gambiaes.l.* following 2 years of PBO-treated net use (2022 vs. 2023)

#### Resistance intensity

**Fig. 5:**
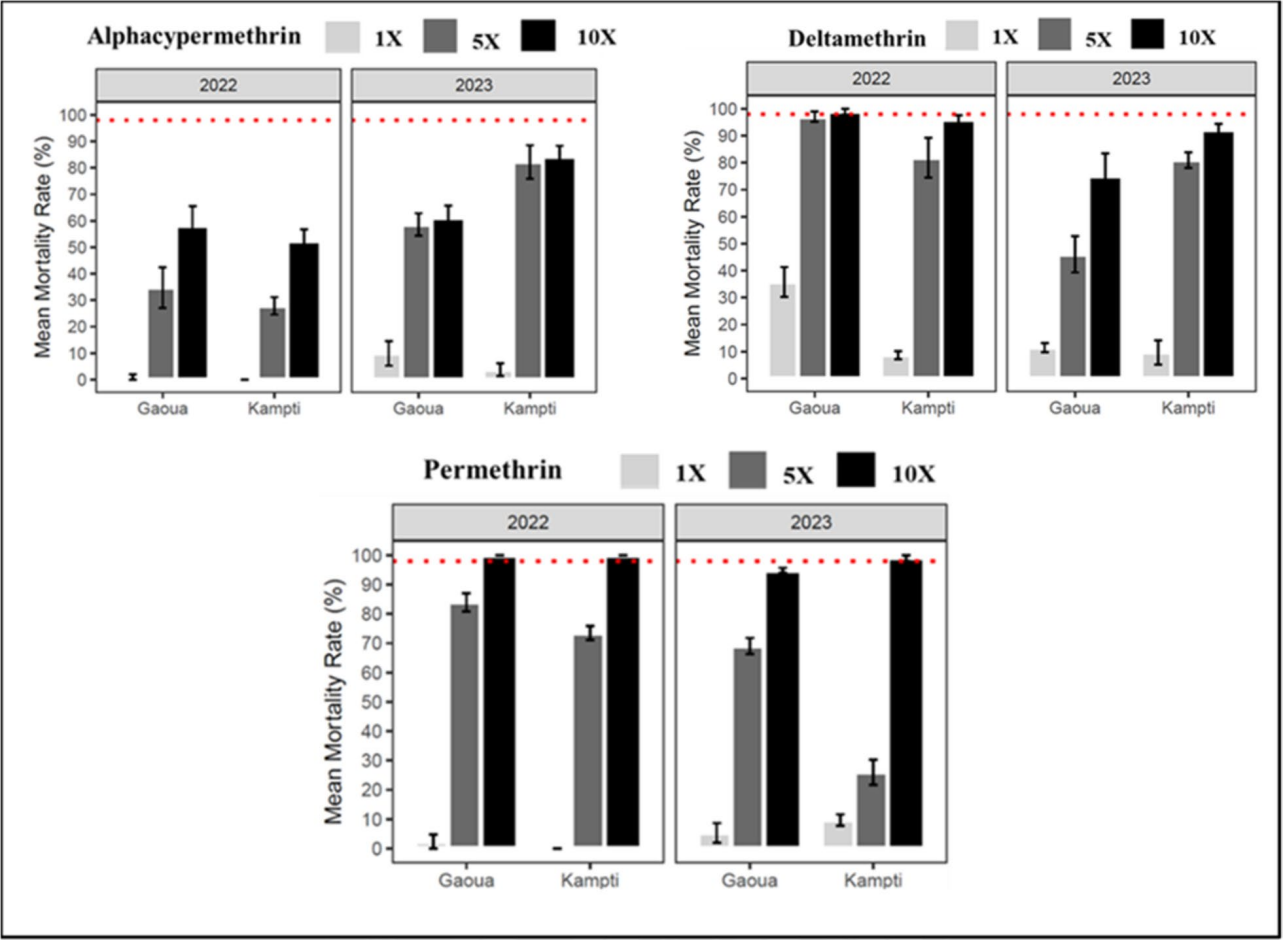
Results of intensity WHO test on *Anopheles gambiae s.l.* in 2022 and 2023 with alpha-cypermethrin (0.05%, 0.25%, 0.50%), deltamethrin (0.05%, 0.25%, 0.50%) and permethrin (0.75%, 3.75%, 7.50%)

The intensity of pyrethroid resistance in *An. gambiae* populations from Kampti and Gaoua was assessed using 1 ×, 5 ×, and 10 × the diagnostic concentrations of deltamethrin, alpha-cypermethrin, and permethrin (Fig. 5). Across the study period, high resistance intensity to alpha-cypermethrin was consistently observed at both sites, as evidenced by mortality rates remaining below the 98% threshold at the 10 × concentration. Resistance intensity to permethrin varied by site and year. Moderate resistance intensity (≥ 98% mortality at 10 × dose) was detected in Gaoua in 2022 and in Kampti in both 2022 and 2023. However, in 2023, high resistance intensity was recorded in Gaoua, with mortality at the 10 × dose reaching only 94.84 ± 1.14% (Supplementary File 3). For deltamethrin, high resistance intensity was observed at all sites in 2022 and 2023, with the exception of Gaoua in 2022, where moderate resistance intensity was recorded (10 × : 98.86 ± 0.86%). Notably, resistance intensity to deltamethrin increased significantly in Gaoua in 2023 (P < 0.0005). In 2022, significant differences in both resistance level (1 × dose) and resistance intensity (5 × dose) to deltamethrin were found between Gaoua and Kampti. In 2023, statistically significant differences in resistance intensity were observed between the two sites for all three insecticides tested (P < 0.001).

#### Synergist bioassay with PBO (4%)

**Fig. 6:**
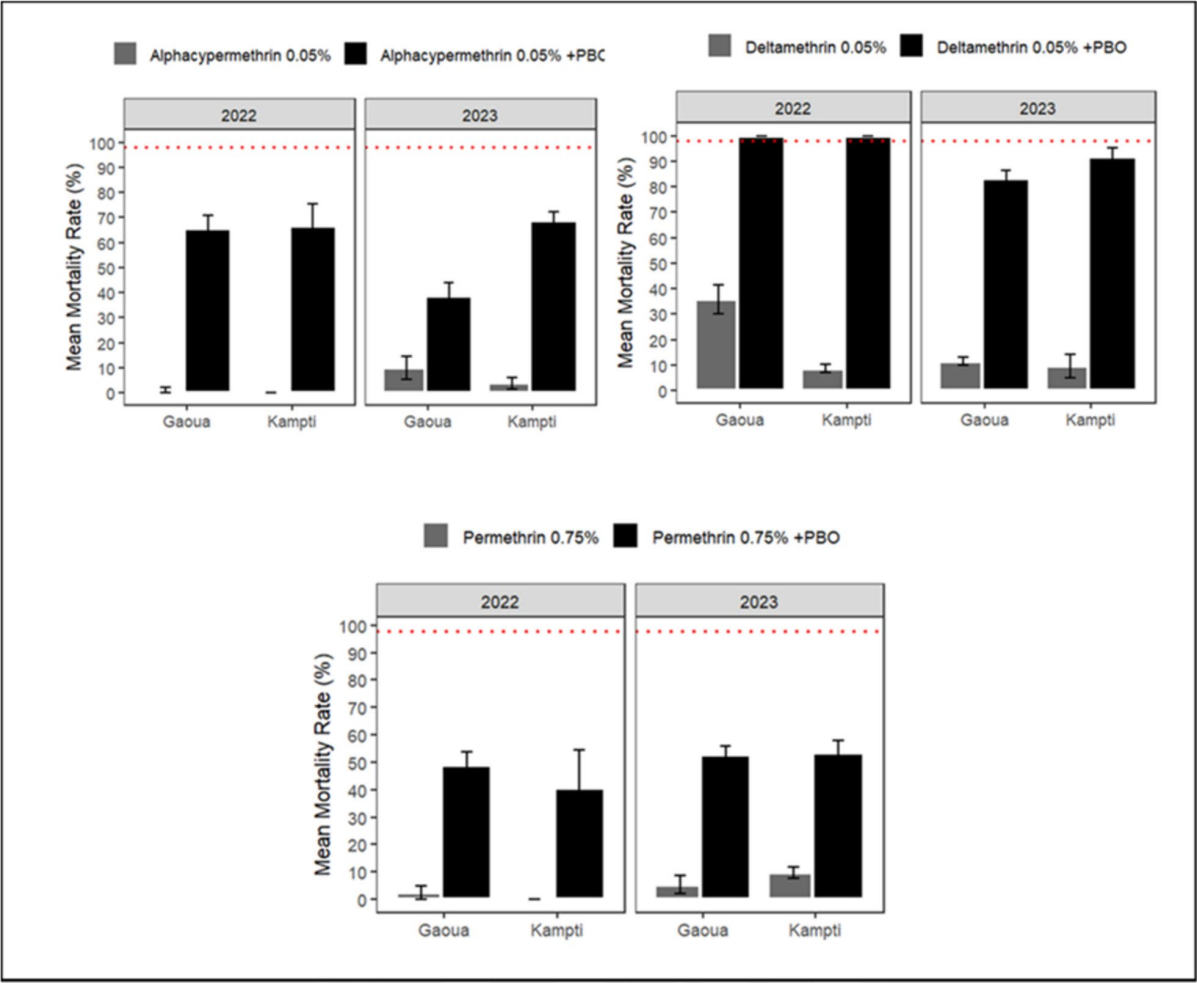
Twenty-four-hour mortality of *Anopheles gambiae s.l.* exposed to alpha-cypermethrin (0.05%), deltamethrin (0.05%), and permethrin (0.75%) with and without PBO pre-exposure in WHO tube tests conducted in 2022 and 2023

Pre-exposure to the synergist piperonyl butoxide (PBO, 4%) followed by exposure to pyrethroids significantly enhanced mortality rates across all tested insecticides deltamethrin (0.05%), alpha-cypermethrin (0.05%), and permethrin (0.75%) at both study sites over the study period (P < 0.01; Fig. 6). However, complete restoration of susceptibility (i.e., 100% mortality) was only achieved in 2022 with deltamethrin at both Gaoua and Kampti. Specifically, mortality following exposure to deltamethrin alone increased from 35.79% ± 5.54% to 100% in Gaoua and from 8.69% ± 1.50% to 100% in Kampti when preceded by PBO pre-exposure. While deltamethrin-only mortality remained unchanged between 2022 and 2023 in Kampti, a significant decline in mortality following PBO pre-exposure was observed in 2023 (X^2^ = 6.1678, df = 1, P = 0.01). No significant differences were found between Gaoua and Kampti in PBO + pyrethroid-induced mortality in either 2022 or 2023, except in 2023 for alpha-cypermethrin. In that year, mortality following PBO pre-exposure and subsequent exposure to alpha-cypermethrin was significantly higher in Gaoua than in Kampti (X^2^ = 16.146, df = 1, P < 0.001).

#### Site-specific overexpression patterns of cytochrome P450s and GSTe2 associated with metabolic resistance in *Anopheles gambiae s.l.* from Southwestern Burkina Faso

**Fig. 7:**
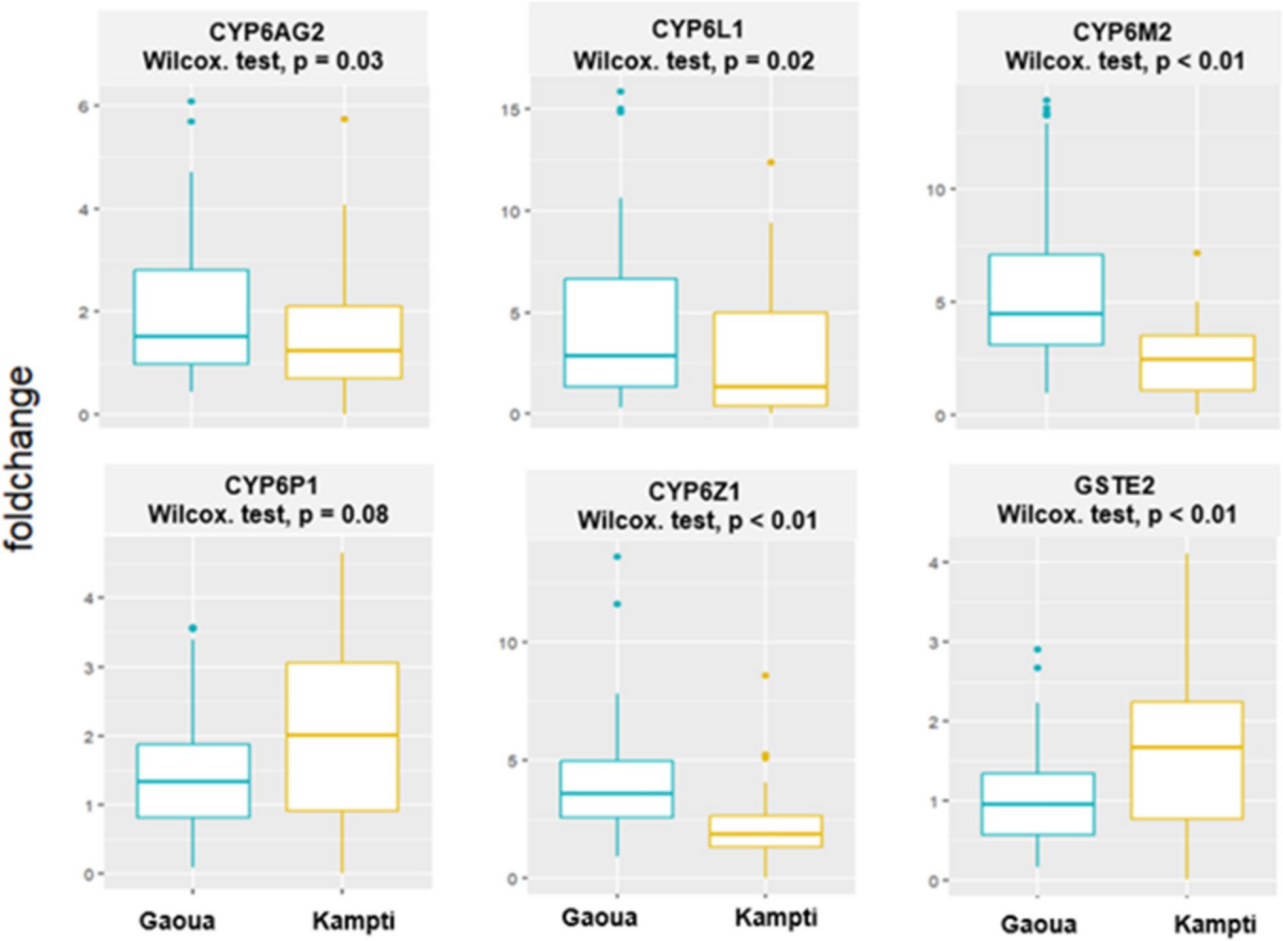
Relative expression levels of candidate detoxification genes in *Anopheles gambiae s.l.* populations from Gaoua and Kampti compared to the susceptible Kisumu reference strain

Gene expression profiling of detoxification enzymes associated with metabolic resistance in *An. gambiae* populations from Gaoua and Kampti (2023) revealed significant overexpression of several cytochrome P450 monooxygenases relative to the insecticide-susceptible Kisumu reference strain (Fig. 7). Expression levels varied across genes and study sites.

Among the tested P450 genes, *CYP6M2* and *CYP6Z1* were significantly overexpressed (fold change > 2) in both populations, with expression levels notably higher in the Gaoua population than in Kampti (P < 0.01). *CYP6L1* was also significantly overexpressed in Gaoua, whereas its expression in Kampti was comparatively lower (P = 0.02). Conversely, *CYP6P1* showed significant overexpression in Kampti, although the difference relative to Gaoua was not statistically significant (P = 0.08). The gene *CYP6AG2* was moderately overexpressed in both populations (fold change between 1 and 2); however, differences in expression compared to the Kisumu strain were not statistically significant. In addition to P450s, the Glutathione S-transferase gene *GSTeE2* was significantly overexpressed in Kampti compared to Gaoua (P < 0.01), suggesting a potential role in the detoxification of pyrethroids or other xenobiotics in this population.

## Discussion

Monitoring the effectiveness of malaria vector control tools remains fundamental for sustaining their protective value and for informing strategic responses to the rapid evolution of insecticide resistance. In Burkina Faso, where malaria transmission remains intense, insecticide-treated nets (ITNs) and indoor residual spraying (IRS) represent the primary pillars of vector control. However, their long-term efficacy is being undermined by the spread of resistance within *An. gambiae* populations. Against this backdrop, the present study provides valuable and timely insights into the spatial and temporal dynamics of insecticide resistance in two high-transmission areas Kampti and Gaoua characterized by different intervention histories. By integrating bioassay, molecular, and gene expression data, this study contributes to a deeper understanding of how resistance mechanisms evolve in response to specific control interventions and environmental pressures. The results reinforce the importance of combining phenotypic and molecular monitoring for a comprehensive assessment of resistance trends. Beyond its national relevance, this study offers regional scientific value for the broader West African context, where resistance to pyrethroids and cross-resistance with other insecticide classes are increasingly threatening malaria control gains. These findings fill critical data gaps for the scientific community and provide the National Malaria Control Programme (NMCP) with operational evidence to guide adaptive resistance management and vector control decision-making.

The species composition of the *An. gambiae* complex observed in this study aligns closely with previous findings from Burkina Faso and other Sahelian regions [17, 18], reaffirming the ecological plasticity of this vector group and its remarkable capacity to adapt to heterogeneous environments. Temporal shifts in species distribution are likely influenced by climatic variations, particularly rainfall and temperature fluctuations, which modify the availability and persistence of larval habitats and consequently shape mosquito abundance and population structure [31, 32]. These ecological dynamics, in turn, modulate the intensity and direction of insecticide selection pressures acting on local vector populations. Susceptibility bioassays conducted between 2021 and 2022 demonstrated widespread and persistent resistance to deltamethrin, alpha-cypermethrin, and permethrin, corroborating earlier reports from Burkina Faso [9, 10] and confirming that pyrethroid resistance remains firmly entrenched in *An. gambiae* populations. The consistently high frequency of the L1014F kdr allele, a pattern well documented across West Africa [33–35], further reinforces this conclusion. However, the lack of a significant difference in *kdr* allele frequency between dead and surviving mosquitoes suggests that target-site resistance alone cannot fully explain the observed phenotypic resistance. The low prevalence of the L1014S mutation indicates a marginal contribution, whereas the near fixation of the L1014F allele even in areas with minimal insecticide use points to a long-term evolutionary stabilization of this mutation within local populations. Molecular and gene expression analyses performed in 2023 revealed strong overexpression of cytochrome P450 genes (CYP6M2 and CYP6Z1) and the glutathione S-transferase GSTeE2, underscoring the growing importance of metabolic resistance mechanisms. The heterogeneity in expression patterns between Gaoua and Kampti parallels previous findings from Côte d’Ivoire that highlighted regional variation in detoxification gene expression associated with local agricultural practices and environmental conditions [36]. Specifically, the overexpression of CYP6L1 in Gaoua and CYP6P1 in Kampti indicates localized adaptive responses to distinct insecticidal pressures. Interestingly, these patterns contrast with earlier data from Gaoua showing lower expression of detoxification genes, suggesting a recent shift in dominant resistance pathways potentially driven by the introduction of pyrethroid–PBO nets. Operationally, the insecticide rotation strategies implemented between 2021 and 2022 did not produce measurable reductions in resistance. In Gaoua, the transition from PermaNet^®^ 2.0 (deltamethrin) to DuraNet^®^ Plus (alpha-cypermethrin + PBO) and in Kampti from PermaNet^®^ 2.0 and IRS with pirimiphos-methyl to Olyset^®^ Plus (permethrin + PBO) were accompanied by declining mosquito mortality and KD60 rates. The stability of kdr frequencies despite these interventions suggests that metabolic resistance mechanisms rather than target-site mutations are sustaining the observed resistance. Moreover, because these interventions relied on insecticides within the same pyrethroid class, they likely maintained similar selection pressures [10, 37]. Pre-exposure to PBO partially restored susceptibility to pyrethroids, with complete restoration observed only for deltamethrin, consistent with reports indicating that deltamethrin + PBO combinations restore susceptibility more effectively than permethrin + PBO [38, 39]. In Gaoua, higher mortality following PBO pre-exposure may be attributed to inhibition of P450 enzymes such as CYP6M2 and CYP6Z1, while in Kampti, the high expression of GSTeE2, which is not inhibited by PBO, likely explains the limited recovery of susceptibility. Elevated GSTeE2 expression in Kampti may also result from prior IRS applications using pirimiphos-methyl, which is known to select for GSTeE2 and induce cross-resistance between pyrethroids and organophosphates [40, 41]. Notably, resistance intensified between 2022 and 2023, as reflected by reduced mortality even after PBO pre-exposure, particularly to deltamethrin, suggesting additive or synergistic selection pressure resulting from the concurrent use of multiple pyrethroid-based interventions. Resistance intensity assays confirmed strong resistance to alpha-cypermethrin and deltamethrin and moderate resistance to permethrin, with the pronounced escalation in deltamethrin resistance in Gaoua emphasizing the value of intensity testing as a complementary tool to standard WHO susceptibility assays. The continuous deployment of pyrethroid-based vector control tools, combined with the agricultural use of similar compounds, likely sustains these resistance trends. Furthermore, the re-use and improper disposal of old ITNs may expose mosquitoes to sub-lethal doses of insecticides, thereby exacerbating selection for resistant phenotypes [42, 43].

The findings of this study have significant implications for both national malaria control programs and the broader scientific community. Notably, they demonstrate that rotation solely within the pyrethroid class is insufficient to curb resistance, emphasizing the need for rotations involving insecticides with distinct modes of action. Furthermore, the partial and inconsistent restoration of susceptibility following PBO pre-exposure indicates that PBO-based nets alone are unlikely to sustainably manage resistance in areas where multiple mechanisms coexist. Consequently, integrating complementary strategies such as next-generation ITNs incorporating dual active ingredients, larval source management, and targeted IRS using non-pyrethroid formulations may diversify selection pressures and enhance control efficacy. For the NMCP of Burkina Faso, these results highlight the importance of incorporating resistance intensity monitoring and molecular diagnostics into routine surveillance to detect early shifts in resistance mechanisms. The observed differences in gene expression profiles between Gaoua and Kampti further underscore the necessity of site-specific management strategies rather than uniform nationwide interventions. Additionally, closer coordination between agricultural pesticide regulation and public health sectors is essential to mitigate cross-sectoral selection pressures. For the scientific community, this study reinforces the value of integrating multi-year, multi-method entomological monitoring with molecular characterization to unravel the complex interplay between operational interventions and resistance evolution. Collectively, such evidence provides a robust foundation for refining global insecticide resistance management frameworks and developing locally tailored, sustainable vector control strategies aimed at preserving the long-term efficacy of malaria interventions in sub-Saharan Africa.

## Conclusions

Building on these findings, this study conclusively demonstrates the dynamic and multifactorial nature of insecticide resistance in *An. gambiae* populations, emphasizing the critical need for continuous and comprehensive surveillance to sustain the efficacy of vector control tools. In the short term, routine monitoring should prioritize the detection of metabolic resistance mechanisms using molecular and biochemical assays, enabling timely, site-specific interventions. Mid-term strategies should focus on elucidating the ecological and genetic determinants of resistance evolution, including the impacts of agricultural pesticide use, urbanization, and gene flow on local vector populations. Long-term approaches must aim to develop sustainable and diversified vector control strategies that reduce reliance on chemical insecticides, incorporating innovations such as next-generation ITNs with dual active ingredients, genetically modified mosquitoes, microbial control agents, and integrated pest management (IPM) frameworks that combine environmental, biological, and chemical control methods. Moreover, research on the operational lifespan and efficacy of next-generation ITNs particularly in relation to insecticide degradation, net aging, and community usage patterns is urgently required to understand their role in resistance dynamics. Importantly, predictive models for malaria control must integrate the potential effects of climate change on vector distribution and resistance evolution. Ultimately, a multi-pronged, adaptive approach informed by real-time phenotypic and molecular surveillance, combined with locally tailored interventions, is essential to mitigate the intensification of resistance and preserve the long-term effectiveness of malaria vector control strategies in Burkina Faso and the broader sub-Saharan African context.

## Supplementary Information

Additional file 1.

Additional file 2.

## Data Availability

No datasets were generated or analysed during the current study.
