# Supplementary figures and images for: Impact of combined vector control interventions on Anopheles gambiae sensu lato resistance dynamics in a high pyrethroid resistance settings in Southwestern Burkina Faso
Source: Malar J. 2025 Dec 5;25:23. doi: 10.1186/s12936-025-05702-1 (PMC12797545; doi:10.1186/s12936-025-05702-1)

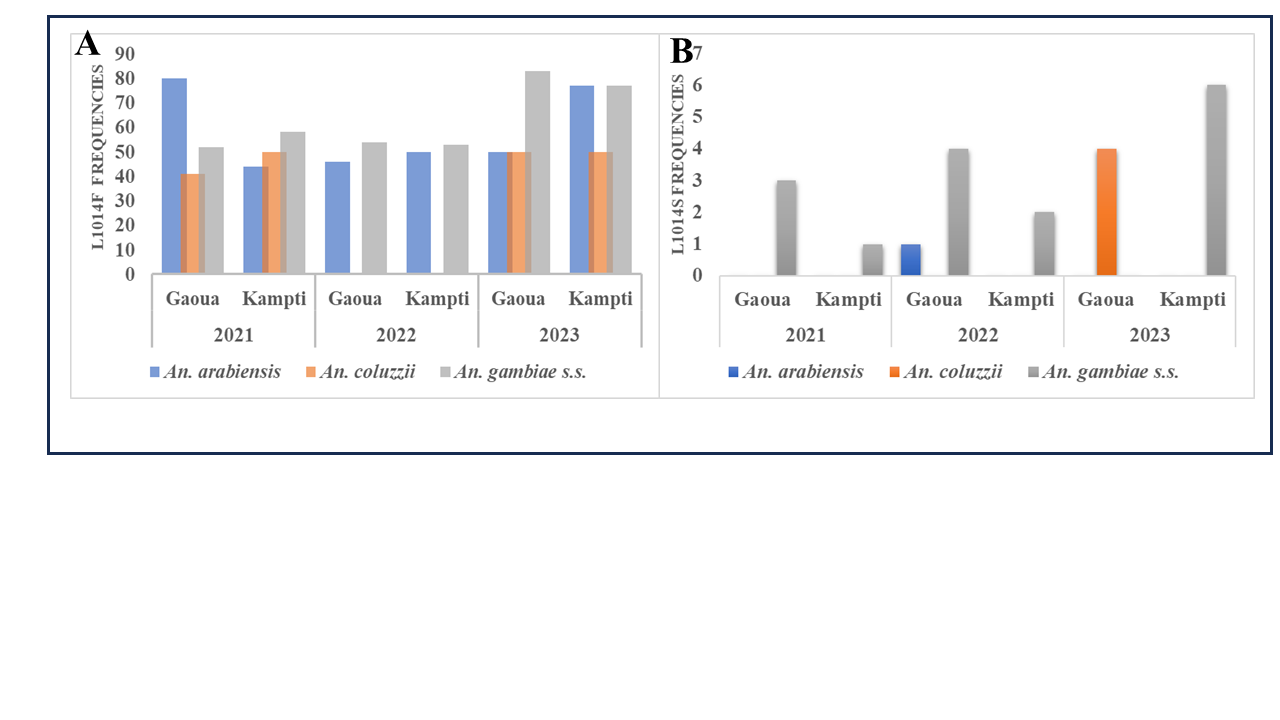

Supplement: Supplementary file 1 — Additional file 1. [file 12936_2025_5702_MOESM1_ESM.tif]
