# Supplementary material for: Impact of combined vector control interventions on Anopheles gambiae sensu lato resistance dynamics in a high pyrethroid resistance settings in Southwestern Burkina Faso
Source: Malar J. 2025 Dec 5;25:23. doi: 10.1186/s12936-025-05702-1 (PMC12797545; doi:10.1186/s12936-025-05702-1)
Supplement: Supplementary file 2 — Additional file 2. [file 12936_2025_5702_MOESM2_ESM.docx]

**Supplementary Files**

**Supplementary File 1**: Species composition of *Anopheles gambiae* s.l. in Gaoua and Kampti from 2021 to 2023.

|  |  |  |  |  |  |  |  |
| --- | --- | --- | --- | --- | --- | --- | --- |
| **Year** | **Site** | ***An. arabiensis*** | | ***An. coluzzii*** | | ***An. gambiae* s.s.** | |
|  |  | **n** | **Proportion (IC 95%)** | **n** | **Proportion (IC 95%)** | **n** | **Proportion (IC 95%)** |
| 2021 | Gaoua | 23 | 23 (15.84 - 32.15) | 8 | 8 (4.11 - 15.00) | 69 | 69 (59.37 - 77.22) |
|  | Kampti | 28 | 28 (20.14 - 37.49) | 1 | 1 (0.18 - 5.45) | 71 | 71 (61.46 - 78.99) |
| 2022 | Gaoua | 46 | 46 (36.56 - 55.73) | 6 | 6 (2.79 - 12.48) | 48 | 48 (38.46 - 57.68) |
|  | Kampti | 12 | 12 (7.00 - 19.81) | 7 | 7 (3.43 - 13.75) | 81 | 81 (72.22 - 87.49) |
| 2023 | Gaoua | 14 | 14 (8.53 - 22.14) | 30 | 30 (21.89 - 39.58) | 56 | 56 (46.21 - 65.33) |
|  | Kampti | 26 | 26 (18.40 - 35.37) | 2 | 2 (0.55 - 7.00) | 72 | 72 (62.51 - 79.86) |
|  |  |  |  |  |  |  |  |

**Supplementary File 3:** Results of WHO resistance intensity tests on *Anopheles gambiae* s.l. in 2022 and 2023 using alpha-cypermethrin (0.05%, 0.25%, 0.50%), deltamethrin (0.05%, 0.25%, 0.50%), and permethrin (0.75%, 3.75%, 7.50%).

| **Site** | **Insecticides** | **Year** | **Mean mortality (SEM)** | **Chi square (χ2)** |
| --- | --- | --- | --- | --- |
| Gaoua | Alphacypermethrin 0.05% | 2022 | 1.00(±1.00) | 0.0207 |
|  |  | 2023 | 9.88(±4.60) |  |
|  | Alphacypermethrin 0.25% | 2022 | 34.76(±7.69) | 0.0019 |
|  |  | 2023 | 58.56(±4.25) |  |
|  | Alphacypermethrin 0.50% | 2022 | 58.08(±7.44) | 0.8270 |
|  |  | 2023 | 61.06(±4.61) |  |
|  | Deltamethrin 0.05% | 2022 | 35.79(±5.54) | 7.658e-06 |
|  |  | 2023 | 11.44(±1.68) |  |
|  | Deltamethrin 0.25% | 2022 | 97.03(±1.91) | 2.231e-14 |
|  |  | 2023 | 46.04(±6.71) |  |
|  | Deltamethrin 0.50% | 2022 | 98.86(±1.13) | 6.243e-06 |
|  |  | 2023 | 75.00(±8.41) |  |
|  | Permethrin 0.75% | 2022 | 2.38(±2.38) | 0.57660. |
|  |  | 2023 | 5.33(±3.30) |  |
|  | Permethrin 3.75% | 2022 | 84.01(±3.07) | 0.0215 |
|  |  | 2023 | 69.02(±2.78) |  |
|  | Permethrin 7.5% | 2022 | 100(±0.00) | 0.0860 |
|  |  | 2023 | 94.84(±0.86) |  |
| Kampti | Alphacypermethrin 0.05% | 2022 | 0.00(±0.00) | 0.2141 |
|  |  | 2023 | 3.75(±2.39) |  |
|  | Alphacypermethrin 0.25% | 2022 | 27.88(±3.28) | 1.773e-14 |
|  |  | 2023 | 82.19(±6.34) |  |
|  | Alphacypermethrin 0.50% | 2022 | 52.38(±4.40) | 3.717e-06 |
|  |  | 2023 | 84.24(±4.05) |  |
|  | Deltamethrin 0.05% | 2022 | 8.69(±1.50) | 0.9419 |
|  |  | 2023 | 9.60(±4.49) |  |
|  | Deltamethrin 0.25% | 2022 | 81.88(±7.35) | 1.0000 |
|  |  | 2023 | 80.92(±2.89) |  |
|  | Deltamethrin 0.50% | 2022 | 95.95(±1.63) | 0.3911 |
|  |  | 2023 | 92.11(±2.24) |  |
|  | Permethrin 0.75% | 2022 | 0.00(±0.00) | 0.9556 |
|  |  | 2023 | 9.68(±1.97) |  |
|  | Permethrin 3.75% | 2022 | 73.48(±2.33) | 1.371e-09 |
|  |  | 2023 | 25.97(±4.25) |  |
|  | Permethrin 7.5% | 2022 | 100(±0.00) | 1.0000 |
|  |  | 2023 | 99.03(±0.96) |  |
|  |  |  |  |  |
